# Greenhead (Tabanus nigrovittatus) *Wolbachia* and Its Microbiome: A Preliminary Study

**DOI:** 10.1128/Spectrum.00517-21

**Published:** 2021-10-13

**Authors:** Emilie Lefoulon, Alex Truchon, Travis Clark, Courtney Long, Daniel Frey, Barton E. Slatko

**Affiliations:** a Molecular Parasitology Group, New England Biolabs, Inc., Ipswich, Massachusetts, USA; University of Nebraska-Lincoln

**Keywords:** *Tabanus nigrovittatus*, *Wolbachia*, greenhead, microbiome

## Abstract

Endosymbiotic *Wolbachia* bacteria are known to influence the host physiology, microbiota composition, and dissemination of pathogens. We surveyed a population of Tabanus nigrovittatus, commonly referred to as “greenheads,” from Crane Beach (Ipswich, MA, USA) for the presence of the alphaproteobacterial symbiont *Wolbachia.* We studied the COI (mitochondrial cytochrome oxidase) marker gene to evaluate the phylogenetic diversity of the studied specimens. The DNA sequences show strong similarity (between 99.9 and 98%) among the collected specimens but lower similarity to closely related entries in the NCBI database (only between 96.3 and 94.7%), suggesting a more distant relatedness. Low levels of *Wolbachia* presence necessitated a nested PCR approach, and using 5 markers (*ftsZ*, *fbpA*, *dnaA*, *coxA*, and *gatB*), we determined that two recognized “supergroups” of *Wolbachia* species were represented in the studied specimens, members of clades A and B. Using next-generation sequencing, we also surveyed the insect gut microbiomes of a subset of flies, using Illumina and PacBio 16S rRNA gene sequencing with barcoded primers. The composition of *Proteobacteria* also varied from fly to fly, with components belonging to *Gammaproteobacteria* making up the largest percentage of organisms (30 to 70%) among the microbiome samples. Most of the samples showed the presence of *Spiroplasma*, a member of the phylum *Mollicutes*, although the frequency of its presence was variable, ranging from 2 to 57%. Another noteworthy bacterial phylum consistently identified was *Firmicutes*, though the read abundances were typically below 10%. Of interest is an association between *Wolbachia* presence and higher *Alphaproteobacteria* representation in the microbiomes, suggesting that the presence of *Wolbachia* affects the host microbiome.

**IMPORTANCE** Tabanus nigrovittatus greenhead populations contain two supergroups of *Wolbachia* endosymbionts, members of supergroups A and B. Analysis of the greenhead microbiome using next-generation sequencing revealed that the majority of bacterial species detected belonged to *Gammaproteobacteria*, with most of the samples also showing the presence of *Spiroplasma*, a member of the *Mollicutes* phylum also known to infect insects. An association between *Wolbachia* presence and higher Alphaproteobacteria representation in the microbiomes suggests that *Wolbachia* presence affects the host microbiome composition.

## INTRODUCTION

*Wolbachia* bacteria are maternally inherited obligate endosymbiotic *Alphaproteobacteria* most closely related to *Ehrlichia* and *Anaplasma*. They are estimated to be present in 40 to 60% of all arthropod species (1, 2) and are classified into 17 clades called “supergroups” (including those within nematodes) (3, 4). In arthropods, they are associated with reproductive manipulations, including feminization, parthenogenesis, male-killing, and cytoplasmic incompatibility, by which they help ensure their own frequency in the population (5–7). The reproductive manipulations have evolutionary consequences, leading to speciation events by reproductive isolation (5, 8–12). While generally thought of as parasitic in arthropods, there is increasing evidence that they have mutualistic benefits for their hosts, for instance in times of nutritional stress or in conferring resistance to viral infections to the host (4, 13–15). As part of a larger phylogenetic survey, we wished to determine the occurrence of *Wolbachia* endosymbionts in Tabanus nigrovittatus.

**FIG 1:**
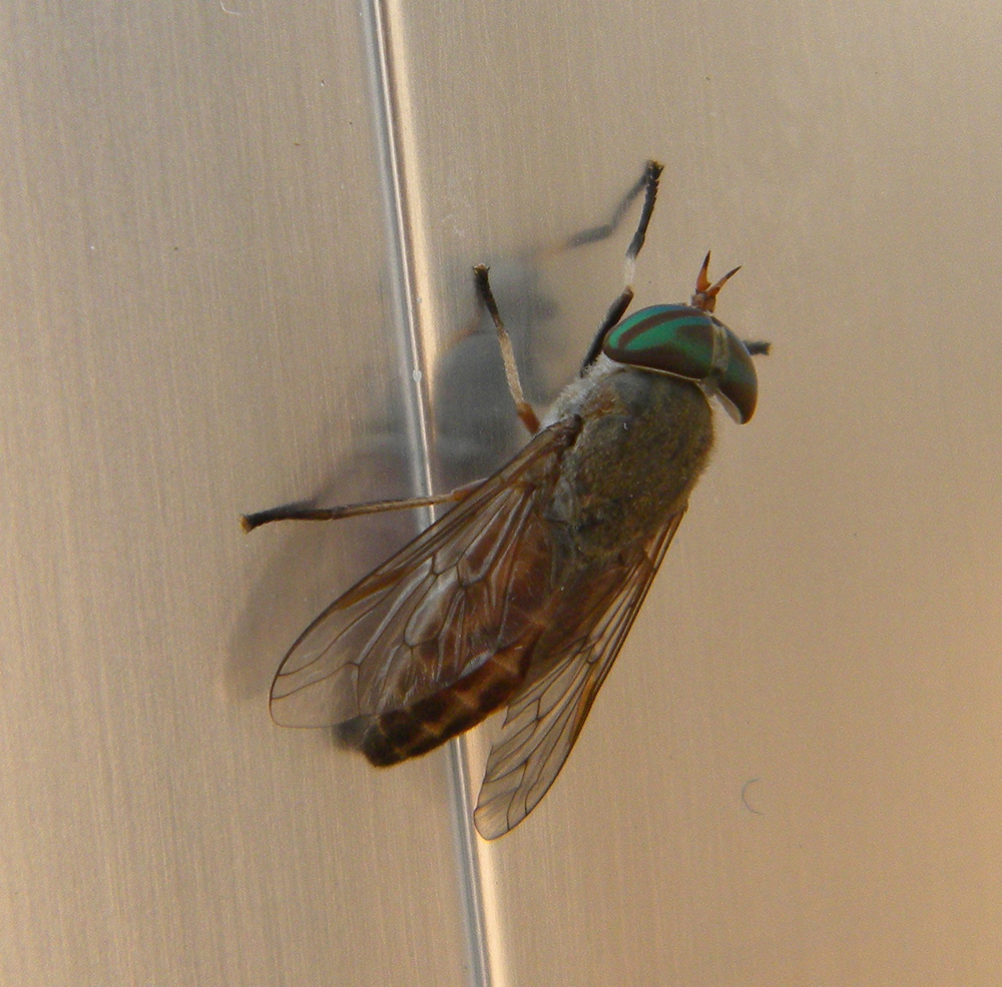
Photo of *Tabanus nigrovittatus* (horsefly), often called “greenhead” due to its large green eyes. Image from https://en.wikipedia.org/wiki/Tabanus_nigrovittatus (https://commons.wikimedia.org/wiki/File:Greenhead_Horse-Fly,_cropped.jpg by Maximilian Paradiz from Merida, Mexico; CC BY 2.0, https://creativecommons.org/licenses/by/2.0, via Wikimedia Commons).

*Tabanus nigrovittatus* horseflies, commonly referred to as “greenheads” due to their large greenish eyes (Fig. 1), are found in the marshes of Massachusetts, as well as in many other locations along the eastern United States (16–18). They belong to a large family of over 140 genera which includes over 4,000 species (19). Greenhead adults typically emerge in midsummer, after larval overwintering and brief pupation in the marshes, often after a salt marsh flooding and usually associated with a corresponding full moon, which has given rise to folklore about their arrival and departure based on the full-moon cycle. Females are the blood-feeding sex, and while the nutritional source for the first egg-laying is derived from larval stage feeding, females require blood meals to produce additional egg masses. Females migrate from the marsh areas to more open landscapes to find blood-meal hosts, including humans. They are notorious pests confounding many outdoor summertime activities, with significant economic effects due to their aggressive blood-feeding using mouthpart structures that create a painful scissor-like piercing to reach blood capillaries (20).

As pests, greenheads are difficult to control, as the majority of their life is spent underground; they surface only once a year and undergo a single generation per season, as opposed to mosquitos, with multiple generations per season. Their wide geographic range, large population size, long-range distance flight ability, and the fact that their eggs and larvae are also a food source for many coastal birds and fish make elimination strategies complicated (17, 18).

Certain *Tabanus* species can transmit diseases and pathogens, in addition to the obvious effects of puncture wounds (19, 21, 22). They are generally not biological vectors of disease but rather mechanical vectors and serve as carriers for pathogens, rather than enabling the pathogens to develop into infective stages within them (19). Diseases carried by tabanids include viruses, bacteria (such as *Anaplasma*), trypanosomes, and filarial parasites (such as Loa loa), which largely infect stock animals such as ruminants and horses. While little is known about human transmission, there is epidemiological evidence that some diseases are carried into humans, such as deer fly fever (caused by Francisella tularensis) (23).

Samples of *Tabanus nigrovittatus* were collected at Crane Beach (Ipswich, MA, USA), adjacent to salt marshes in late July 2019, to evaluate the presence and local phylogenetic diversity of the *Wolbachia* endosymbiont. We further analyzed the collected samples to determine their intestinal microbiome diversity.

## RESULTS

### *Tabanus nigrovittatus* diversity.

**FIG 2:**
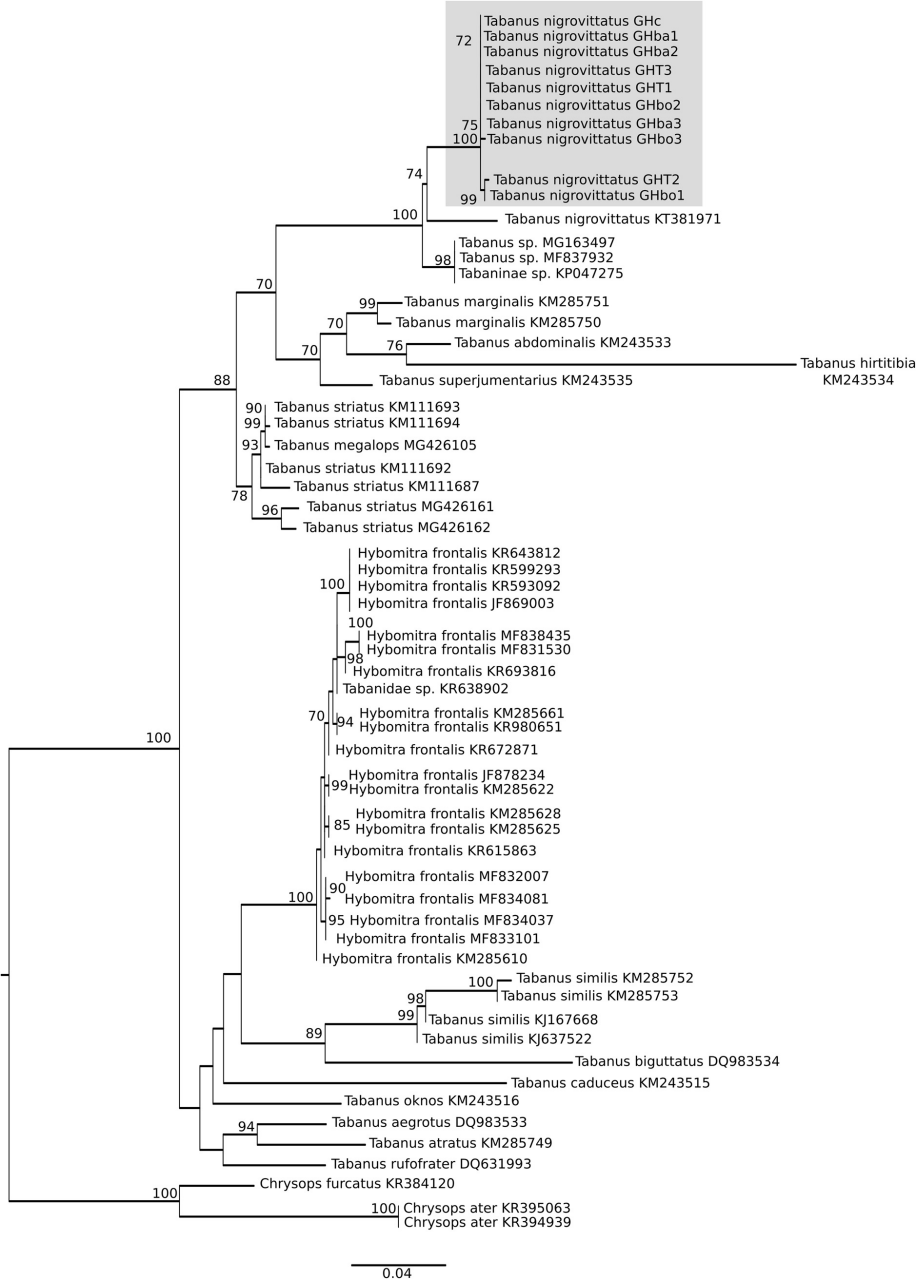
Phylogeny of the COI gene of *Tabanidae* species. The total length of the data sets is 614 bp. The topology was inferred using maximum likelihood (ML) inference using IQ-TREE. The best-fit model, calculated using ModelFinder according to the Bayesian information criterion (BIC) index, was TIM3+I+G4. The nodes are associated with bootstrap values based on 1,000 replicates; only bootstrap values over 70 are shown.

Phylogenetic analysis of COI (mitochondrial cytochrome oxidase) was performed to study the relationships among the collected *Tabanus nigrovittatus* specimens, as well as their relationships with species belonging to the genera *Tabanus* and *Hybomitra* (Fig. 2). The COI analysis showed that all 10 of the collected *T. nigrovittatus* specimens form a clade and are closely related (Fig. 2). The pairwise comparisons of the COI marker were between 99.9 and 98% similar for the collected specimens (Table S4 in the supplemental material). However, when we studied the pairwise comparison between the collected specimens and the only COI sequence belonging to *Tabanus nigrovittatus* available at NCBI (GenBank accession number KT381971), we observed only between 96.3 and 94.7% similarity (Table S4 in the supplemental material). Our specimens are closely related but are more distant from the available reference (Fig. 2). The produced phylogeny of COI (Fig. 2) indicates that all the COI sequences of *Tabanus nigrovittatus* specimens form one clade. This analysis of COI sequences does not show different groups among the studied samples, suggesting that we are not studying different populations of *T. nigrovittatus*.

### Detection and characterization of *Wolbachia* infection.

For the detection of *Wolbachia* in *T. nigrovittatus* by PCR amplification, we consistently observed very little amplification using a standard PCR protocol. Positive *Wolbachia* signals were only detected after using nested a PCR protocol to amplify the signals from previous PCR products (24). Based upon previous observations, these data suggest that the levels of *Wolbachia* colonization are likely low. Further, we found that only 6 of 10 specimens were positive for *Wolbachia* in our nested PCR assay. While these data suggest a 60% colonization rate, more rigorous sampling would be required to firm up this estimate. It is not unusual for populations to have a variable number of infected individuals and/or for individuals to have low *Wolbachia* titers (25–27). As a further validation of the nested PCR assay, positive PCRs were sequenced by Sanger sequencing and determined to be *Wolbachia* through sequence comparisons.

**FIG 3:**
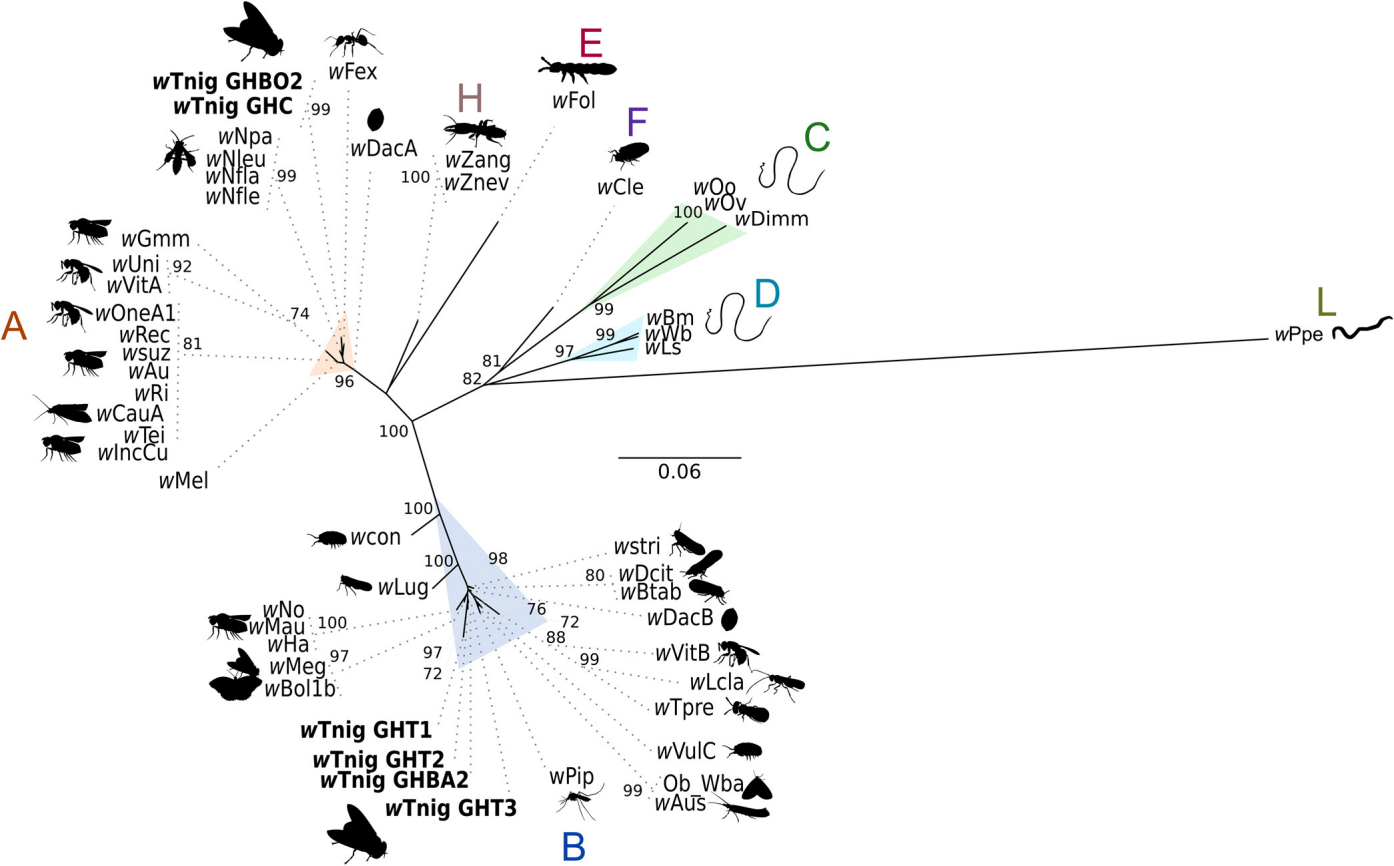
Unrooted phylogeny of *ftsZ* genes of *Wolbachia*. The total length of the data sets is 616 bp. The topology was inferred using maximum likelihood (ML) inference using IQ-TREE. The best-fit model, calculated using ModelFinder according to the BIC index, was TN+G4. The nodes are associated with bootstrap values based on 1,000 replicates; only bootstrap values over 70 are shown.

Only the *ftsZ* marker was successfully amplified and sequenced for the entire set of *Wolbachia*-positive specimens. The phylogeny based on *ftsZ* showed two types of infection: four specimens (T1, T2, T3, and BA2) harbored a *Wolbachia* symbiont belonging to *Wolbachia* supergroup B, and two specimens (C and BO2) harbored a symbiont belonging to *Wolbachia* supergroup A (Fig. 3). Analyses of four additional markers (*dnaA*, *fbpA*, *gatB*, and *coxA*) provided similar results (Fig. S1 in the supplemental material). Indeed, the analysis of *dnaA* confirmed that specimens C and BO2 harbored *Wolbachia* supergroup B, and specimen BA2 harbored *Wolbachia* supergroup A. The analysis of *fbpA* confirmed that specimen C harbored *Wolbachia* supergroup B, and specimens T1 and BA2 harbored *Wolbachia* supergroup A. The analysis of *coxA* confirmed that specimen BO2 harbored *Wolbachia* supergroup B, and specimen BA2 harbored *Wolbachia* supergroup A. Finally, the analysis of *gatB* only confirmed that specimen C harbored *Wolbachia* supergroup B.

### Microbiomes of *Tabanus nigrovittatus*.

**FIG 4:**
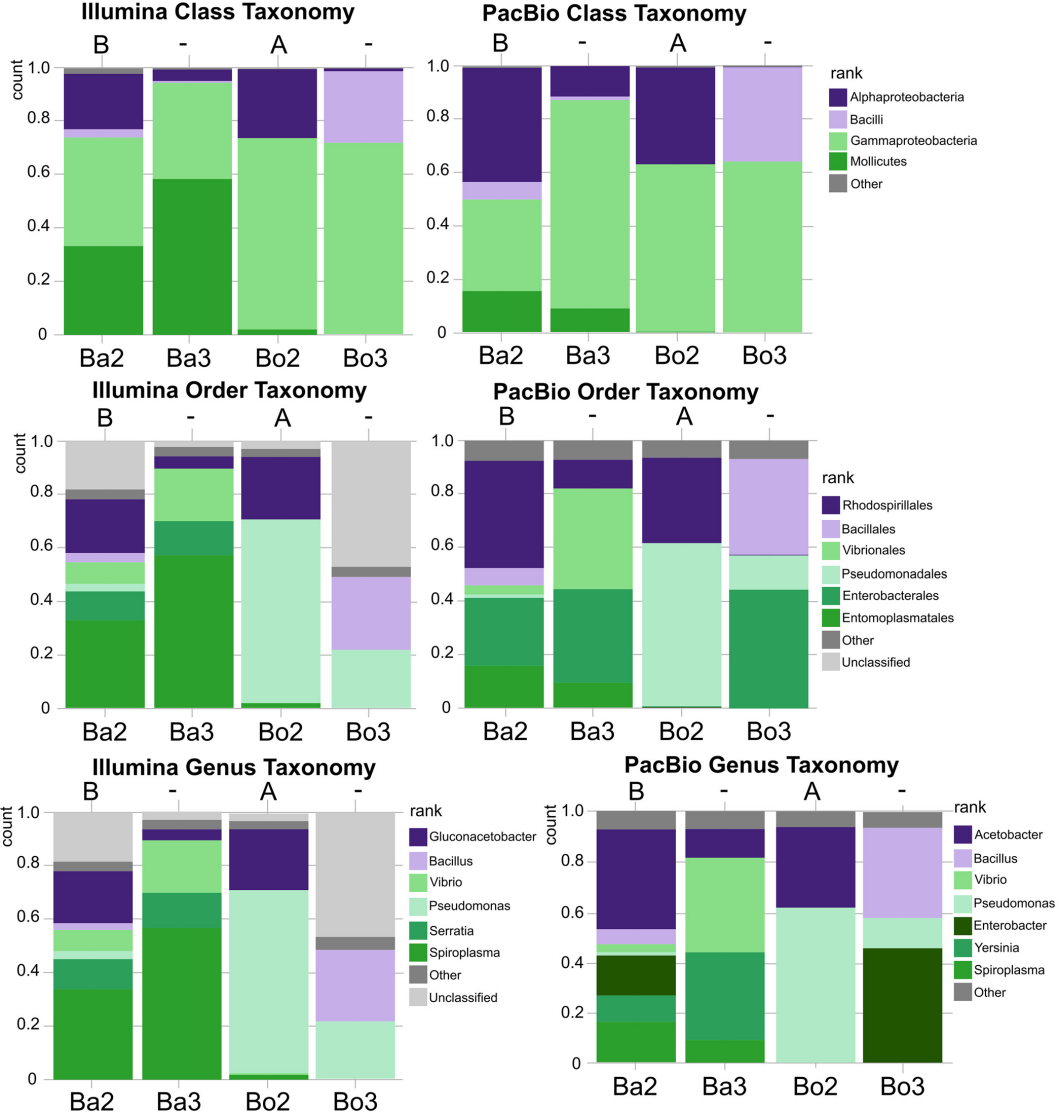
Microbiomes of individual *T. nigrovittatus* flies, as defined at the class, order, and genus levels. The resulting population statistics were based on the Ribosomal Database Project definition. B, presence of *Wolbachia* from supergroup B; A, presence of *Wolbachia* from supergroup A; –, no *Wolbachia* presence detected by PCR.

We examined the microbiomes of four specimens of *T. nigrovittatus* (BA2, BA3, BO2, and BO3) by preparing and sequencing the 16S rRNA gene using Illumina (V4 region) and PacBio (full-length 16S rRNA gene amplicon) technologies (Table S5 in the supplemental material, Fig. 4). In general, both the Illumina and PacBio sequenced data were fairly consistent for individuals BO2 and BO3. More variability was observed for BA2 and BA3. For both of them, PacBio sequencing indicated more *Alphaproteobacteria* and *Gammaproteobacteria* presence than with Illumina sequencing, while the Illumina data showed a higher presence of *Mollicutes* than the PacBio sequencing (Table S5 in the supplemental material, Fig. 4). Generally, the samples sequenced using the Illumina NextSeq system showed a higher *Spiroplasma* presence than in the PacBio Sequel data (Table 1 and Fig. 4). This may be the result of biases in either the library preparation or the different PCR primers used before sequencing. There were few other differences between the sampled populations, as all microbiomes consisted mainly of *Gammaproteobacteria*, *Alphaproteobacteria*, *Mollicutes*, and *Bacillus*. PacBio sequencing libraries were also prepared for six other individual flies, although the quality of these PCR-based libraries was significantly inferior to those of the four that were sequenced on both platforms, and they were not included in the study. These produced sequences were also relatively consistent among individuals (data not presented).

**TABLE 1:** Percentage of each genus and family determined for four samples, BA2, BA3, BO2, and BO3

Genus	Phylum	Order	Sequencing technology	Content (%) for sample^*a*^:			
				BA2 (W+)	BA3 (W−)	BO2 (W+)	BO3 (W−)
Pseudomonas	*Gammaproteobacteria*	*Pseudomonadales*	Illumina	3	<0.01	69	22
			PacBio	2	<0.01	62	13
*Vibrio*	*Gammaproteobacteria*	*Vibrionales*	Illumina	8	20	<0.01	<0.01
			PacBio	3	38	<0.01	<0.01
Enterobacter	*Gammaproteobacteria*	*Enterobacterales*	Illumina	ND	ND	ND	ND
			PacBio	16	<0.01	<0.01	45
*Yersinia*	*Gammaproteobacteria*	*Enterobacterales*	Illumina	ND	ND	ND	ND
			PacBio	10	35	<0.01	<0.01
*Serratia*	*Gammaproteobacteria*	*Enterobacterales*	Illumina	11	13	<0.01	<0.01
			PacBio	ND	ND	ND	ND
*Acetobacter*	*Alphaproteobacteria*	*Rhodospirillales*	Illumina	ND	ND	ND	ND
			PacBio	40	11	32	<0.01
*Gluconacetobacter*	*Alphaproteobacteria*	*Rhodospirillales*	Illumina	20	4	23	<0.01
			PacBio	ND	ND	ND	ND
*Spiroplasma*	*Mollicutes*	*Entomoplasmatales*	Illumina	33	57	2	<0.01
			PacBio	16	9	<0.01	<0.01
*Bacillus*	*Firmicutes*	*Bacillales*	Illumina	3	<0.01	<0.01	27
			PacBio	6	<0.01	<0.01	36
Unclassified			Illumina	18	2	3	47
			PacBio	ND	ND	ND	ND
Other			Illumina	4	4	3	5
			PacBio	6	8	6	6

a W+ and W− refer to *Wolbachia* presence and absence, respectively, based upon PCR analysis. ND, not detected. Color code: 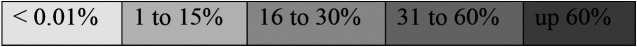

The microbial composition of *T. nigrovittatus* is primarily defined by a limited diversity of bacterial phyla. In most cases, *Proteobacteria* is the most dominant phylum within the microbiome, as was noted in multiple specimens. In most cases, the majority belong to the class *Gammaproteobacteria*, which made up the majority of classified reads, except in one sample (BA3), which also contained high numbers of *Spiroplasma* (*Mollicutes*) Illumina sequence reads (in contrast to the PacBio sequencing result, as described previously). Within the *Gammaproteobacteria*, a wealth of *Vibrio*-related ribosomal reads in two high-quality samples (BA2 and BA3) were consistent with those of Vibrio fluvialis and Vibrio furnissii. They composed 3 and 8% of the microbiome of BA2 (based on PacBio and Illumina sequencing, respectively) and 20 and 38% (based on Illumina and PacBio sequencing, respectively) of the microbiome of BA3, while <0.01% were detected for BO2 and BO3 (Table 1). *Vibrio* was also identified in one low-quality microbiome in which it composed 25% of reads (data not presented).

For samples BO2 and BO3, most of the *Gammaproteobacteria* reads were identified as Pseudomonas, while low abundances were detected for BA2 and BA3 (Table 1). As another representative of *Gammaproteobacteria*, *Enterobacterales* are detected in most microbiomes (with the exception of BO2) (Fig. 4). Interestingly, the PacBio analysis suggested the presence of *Yersinia* and Enterobacter, while the Illumina analysis identified only *Serratia* (Fig. 4, Table 1). In addition, a high number of unclassified read at the genus level was observed for the Illumina sequencing (in particular, for BA2 and BO3) (Fig. 4).

With respect to *Alphaproteobacteria* present in the microbiomes, *Rhodospirillales* are the most abundant (*Acetobacter* or *Gluconacetobacter*) that are likely acetic acid-producing bacteria (Fig. 4). Of interest is that for the two *Wolbachia-*positive individuals (BA2, harboring *Wolbachia* from supergroup B, and BO2, harboring *Wolbachia* from supergroup A), we observed a tendency toward higher counts of *Alphaproteobacteria* reads (21 to 25% for PacBio sequencing; 37 to 44% for Illumina sequencing) than those within *Wolbachia*-negative individuals (1 to 4% for PacBio; 1 to 11% for Illumina) (Table S5 in the supplemental material, Fig. 4). Analysis of the PacBio reads suggests the presence of *Acetobacter*, while analysis of the Illumina reads shows the presence of *Gluconacetobacter*, as majority representatives of *Alphaproteobacteria* (Table 1, Fig. 4). Although they represent less than 1% of the microbiome, *Bacteroidetes* and *Actinobacteria* are also present in each microbiome sample. These representatives are typically either classified as anaerobic bacteria associated with the gut microbiome (e.g., *Bifidobacterium* and *Bacteroides*) or aerobic bacteria associated with soil (e.g., *Arthrobacter*).

As mentioned, most of the samples showed the presence of *Spiroplasma* (*Mollicutes*) (except for BO3, which showed <0.01%) (Table 1). The presence of *Spiroplasma* species, and more specifically, Spiroplasma litorale, in greenheads has been previously described (28, 29). In two samples (BA2 and BA3), its presence was higher than in the other two samples (BO2 and BO3). Whether the higher occurrence was due to its high occurrence in the microbiome while the fly was alive or opportunistically after the death of the fly is unknown.

Besides the presence of *Spiroplasma*, the other noteworthy bacterial genus consistently identified was *Bacillus* (bacilli), although the read abundance was typically below 10%, except for one sample (BO3), which showed high abundances (27 to 36%) (Table 1, Fig. 4).

## DISCUSSION

This is the first observation of *Wolbachia* in *T. nigrovittatus*. Based on our phylogenetic analyses, we identified *Wolbachia* endosymbionts from two different supergroups, A and B, harbored by *T. nigrovittatus*. It is not unusual for different *Wolbachia* supergroups to be present in the same arthropod populations or individuals (30–40). Thus, it is possible that there are two lineages infecting this population of flies. There is, however, no evidence of cohabitation between the diverging *Wolbachia* lineages within *T. nigrovittatus* individuals.

One interesting observation concerns a potential effect of *Wolbachia* endosymbiosis on the presence of *Alphaproteobacteria* in individuals. Of the four high-quality microbial 16S rRNA amplicon analyses that were performed, the two individuals that tested positive for *Wolbachia* (either supergroup A or B) both presented a tendency toward higher counts of *Alphaproteobacteria* representation in their microbiomes (Fig. 4). The presence of *Wolbachia* might favor colonization by *Alphaproteobacteria* or vice versa. However, the dominant *Alphaproteobacteria* identified here were not *Wolbachia* species, nor did they fall within the intracellular *Rickettsiales*; rather, they were almost entirely acetic acid *Rhodospirillales* bacteria. While all the low-quality microbiomes which tested positive for *Wolbachia* had dominant acetic acid bacteria populations, there was not a significant increase from the other low-quality 16S rRNA microbiomes. Although the evidence provided here is limited to just four individuals, the findings support the case of *Wolbachia* endosymbionts affecting an organism’s microbiome.

The *T. nigrovittatus* microbiome is very much akin to that of many similar arthropods, especially other *Diptera* (41), although the abundance of its various representatives is quite variable from fly to fly. *Gammaproteobacteria* have been described as frequently dominating the microbiomes of arthropods (41). For two of our samples, we observed either a high presence of Pseudomonas (BO2) or Enterobacter (BO3). The two other samples presented multiple *Gammaproteobacteria*: *Vibrio*, *Yersinia* associated with Enterobacter, and Pseudomonas (in the case of BA2).

*Spiroplasma* bacteria are common in blood-feeding arthropods, where they can be commensalists, mutualists, or more rarely, pathogens (42–44). While the association may be of an inherited mutualism, the high incidence of *Spiroplasma* in some samples (BA2 and BA3) and not in others (BO2 and BO3) may rather be associated with the different population sampling. While it was recently shown that the presence of *Spiroplasma* leads to reduced *Wolbachia* titers in quill mites (45), our results do not show the same tendency. Of the two individuals having a higher level of *Spiroplasma*, one was infected by *Wolbachia* bacteria (BA2) but not the other (BA3). However, our sample (four individuals) was too small to form a strong conclusion.

Differences in classification between short-read partial 16S-based microbiomes and long-read full gene-based microbiomes were observed and coincide with previous studies analyzing community structure (44, 46, 47). While our Illumina primers targeted the V4 region of the bacterial 16S rRNA subunit, comparison to the whole-gene amplification of PacBio single-molecule real-time (SMRT) sequencing suggests that short reads may miss important elements of microbial diversity. Our data show that Illumina data analysis provides a less effective taxonomic profiling than PacBio data analysis, with a high percentage of unclassified reads at the order and genus levels (particularly for BA2 and BO3). The mischaracterization of the genus *Acetobacter* as *Gluconacetobacter* is a fairly minute detail; both are acetic acid bacteria and are classified accurately down to the family level. The inability to taxonomically classify Enterobacter may result in large discrepancies in microbial abundance. Ultimately, while Illumina short-read sequencing is effective in microbiome analysis at a higher taxonomic level, long-read analyses show much greater power for in-depth classification (44, 46, 47).

## CONCLUSIONS

Based upon the PCR and sequencing analysis performed on *T. nigrovittatus* individuals, *Wolbachia* endosymbionts from both supergroups A and B are present in the sampled population, at a frequency of about 60%, estimated from the small sample size. We have no evidence that more than one supergroup is present in any one individual or that the presence of any one particular supergroup relates to the any of the sampling. The *T. nigrovittatus* microbiome shares commonality with other *Diptera* microbiomes, consisting of *Gammaproteobacteria*, *Alphaproteobacteria*, *Mollicutes*, and *Firmicutes*, although the abundances of its representatives are quite variable from fly to fly. All populations also contain *Spiroplasma* at various levels. Although the evidence provided here is limited to just four individuals, the findings support the supposition of an interaction between *Wolbachia* endosymbionts and an organism’s microbiome, especially *Alphaproteobacteria*. This preliminary report extends the presence of *Wolbachia* to a new species and provides a set of protocols for similar analyses in other organisms.

## MATERIALS AND METHODS

### Sampling and DNA extraction.

Female (biting) greenheads were hand collected at several locations at Crane Beach (managed by the Trustees of Reservations) in Ipswich, MA, in the summer of 2019 and stored on ice until being frozen at −20°C. For DNA isolation, the flies were surface sterilized by immersion in a 50% bleach solution (4% sodium hypochlorite) for 5 min and rinsed three times with a large volume of sterile water in petri dishes. DNA was isolated using the Monarch DNA isolation kit (New England Biolabs, NEB), after first cutting the flies into small pieces on a petri dish on ice, removing the heads and wings, and then grinding them in a small sterile mortar and pestle on dry ice. The ground material was transferred to lysis buffer in 1.5-ml microcentrifuge tubes, and 10 mg/ml proteinase K was added before overnight incubation at 56°C with shaking in a thermomixer at 800 rpm. The procedures and volumes followed those described in the NEB kit manual (for tissue isolation), and DNA was eluted from the column using 100 μl of elution buffer. The yields were somewhat variable among the individual isolates but provided sufficient DNA for both PCR analysis and microbial 16S rRNA amplicon sequencing analysis. The quality of the DNA was analyzed on 1% agarose gel and quantified using a Qubit fluorometer (Invitrogen).

### Greenhead characterization.

A total of 10 specimens were selected for the study from 3 independent samplings (on different dates) in 2019 from Crane Beach (Ipswich, MA; managed by the Trustees of Reservations). We analyzed the specimens labeled GHC and GHBO1 to GHBO3 for the first batch, GHBA1 to GHBA3 for the second batch, and GHT1 to GHT3 for the third. In order to verify the quality of the DNA extraction and study the diversity of the sampling, PCR targeting the host COI (mitochondrial cytochrome oxidase) gene was performed. The PCR amplification was performed using the broad-range DNA primers LCOI490 (5′-GGTCAACAAATCATAAAGATATTGG-3′) and HC02198 (5′-TAAACTTCAGGGTGACCAAAAAATCA-3′) (48). PCR was performed in a final volume of 25 μl: 1× OneTaq buffer (MgCl_2_-free), 2.5 mM MgCl_2_, 0.3 mM of each deoxynucleoside triphosphate (dNTP), 0.5 μM of each primer, and 0.625 units of OneTaq Hot Start DNA polymerase (NEB). The thermal profile was as follows: 94°C for 3 min; 38 cycles of 94°C for 30 s, 42°C for 45 s, and 68°C for 90 s; then, 68°C for 5 min. For all PCRs, negative controls (no DNA) were also performed to rule out contamination artifacts. A total of 10 COI PCR products were Sanger sequenced on an ABI 3730 automated DNA sequencer at the NEB DNA Sequencing Core Facility (Table S1 in the supplemental material). The sequences were deposited in the GenBank data library under the accession numbers MN919538 to MN919547 (Table S1 in the supplemental material). The produced sequences were compared with closely related species (*Tabanus* spp. and *Hybomitra* spp.), selected based on BLASTn analysis.

### Detection and molecular characterization of *Wolbachia* symbionts.

Prescreening for *Wolbachia* presence was first performed by nested PCR amplification of the *Wolbachia*-specific *ftsZ* (cell division) gene using the primer pair ftsZF3/R3 (5′-GCAAATACYGATGCTCARGC-3′ and 5′-ATCAATRCCAGTTGCAAGAA-3′), followed by ftsZF4/R4 (5′-CTAAGGGDCTTGGTGCTGGT-3′ and 5′-ACYTCTTCRCGCACTCTATT-3′). Four other genes were also amplified (*dnaA*, *coxA*, *fbpA*, and *gatB*), as described by Lefoulon et al. (24) (Table S2 in the supplemental material). For all PCRs, negative controls (no DNA) were also performed to rule out contamination artifacts. The PCR products were Sanger sequenced after purification using the NEB Monarch PCR purification kit. The sequences were analyzed by comparison with available sequences extracted from *Wolbachia* complete or draft genome sequences and the addition of sequences from *Wolbachia* from *Zootermopsis angusticollis* and *Zootermopsis nevadensis* (Table S3 in the supplemental material).

### Phylogenetic analyses.

Ortholog sequence alignments were generated using MAFFT (49). For the multilocus phylogenies, a supermatrix of the alignments was generated using SeaView (50). The phylogenetic analyses were performed with maximum likelihood inference using IQ-TREE (51). The most appropriate model of evolution was evaluated using ModelFinder (52) (implemented as a functionality of IQ-TREE). The robustness of each node was evaluated by a bootstrap test (1,000 replicates). Regarding the phylogeny of COI, sequences from *Chrysops* spp. were added as an outgroup. The phylogenetic trees were edited using FigTree (53) and Inkscape (54).

### Microbial 16S rRNA amplicon library preparation for Illumina sequencing.

For 4 specimens, the 16S rRNA gene was amplified using the following pair of primers: the primer 515F (5′-AAT GAT ACG ACC ACC GAG ATC TAC ACT ATG GTA ATT GTG TGC CAG CMG CCG CGG TAA-3′) and the barcoded 806RC primer (5′-CAA GCA GAA GAC GGC ATA CGA GAT [12 nucleotide Illumina barcode] AGT CAG TCA GCC GGA CTA CHV CGG TWT CTA AT-3′). PCRs were performed in a final volume of 25 μl of the following mixture: 1× Q5 Hot Start high-fidelity master mix (NEB), 0.2 μM of each primer, and 100 ng DNA. The thermal profile was as follows: 94°C for 3 min; 25 cycles of 94°C for 45 s, 50°C for 60 s, and 72°C for 90 s; then, 72°C for 10 min. For each specimen, triplicate PCR amplifications were performed, and the DNA was gel purified using the Monarch DNA gel extraction kit (NEB). The DNA samples were eluted in 20 μl 0.1× Tris-EDTA (TE) and quantitated on the Agilent Bioanalyzer system using the DNA 1000 chip according to the manufacturer’s instructions. The Illumina sequencing was performed following the standard Illumina NextSeq protocols using the following primers: Read 1 (5′-TAT GGT AAT TGT GTG CCA GCM GCC GCG GTA A-3′), Read 2 (5′-AGT CAG TCA GCC GGA CTA CHV GGG TWT CTA AT-3′), and index (5′-ATT AGA WAC CCB DGT AGT CCG GCT GAC TGA CT-3′).

### Microbial 16S rRNA amplicon library preparation for PacBio sequencing.

For 10 specimens, the 16S rRNA gene was amplified using the following pair of primers: 27F (5′-GGT AG [16 nucleotide barcode] AGR GTT YGA TYM TGG CTC AG-3′) and 1459R (5′-GGT AG [16 nucleotide barcode] RGY TAC CTT GTT ACG ACT T-3′). PCRs were performed in a final volume of 25 μl of the following mixture: 1× Q5 Hot Start high-fidelity master mix (NEB), 0.3 μM of each primer, and 50 ng DNA. The thermal profile was as follows: 98°C for 30 s; 30 cycles of 98°C for 20 s, 68°C for 30 s, and 72°C for 60 s; then, 72°C for 2 min. The PCR products were determined on the Agilent Bioanalyzer system using the DNA 12000 chip according to the manufacturer’s instructions. The barcoded samples were then pooled to reach an equimolar amount and purified using AMPure PB beads (0.8× ratio of beads). A PacBio SMRTbell library was prepared using the SMRTbell Express template prep kit v2.0 (PacBio), following the manufacturer’s instructions, with the difference that all the AMPure PB bead purifications were performed using a 0.8× bead ratio. The SMRTbell library was then sequenced on the PacBio Sequel system using the diffusion protocol.

### Analysis of amplicon sequencing.

For analysis of the microbial 16S rRNA amplicon sequencing results, the PacBio reads were demultiplexed using seqtk (55). The Illumina read quality was assessed and the reads were trimmed using Trim Galore (using the following parameters: –phred33 –fastqc –illumina –clip_R1 5 –three_prime_clip_R2 5), and low-quality reads were removed from the data set. Analysis of the Illumina sequence quality was performed using the Qiime2 pipeline (56) in order to attain representative sequences from each processed fly and construct a phylogenetic tree, producing diversity metrics. In order to similarly analyze the Illumina and PacBio data, classification of reads was performed based on the Ribosomal Database Project (RDP) 16S small subunit rRNA database (57) using the default settings for the Kraken 2 taxonomic sequence classification system (58).

### Data availability.

The data generated are available in GenBank under the BioProject accession number PRJNA600244 and the BioSample accession number SAMN13810039. The raw data are available in the GenBank Sequence Read Archive (SRA) under the accession numbers SRR10868767 to SRR10868779. The COI sequences are available in GenBank under the accession numbers MN919540 to MN919547. The *Wolbachia* sequences are available in GenBank under the accession numbers MN937243 to MN937257.
