# Supplementary material for: Greenhead (Tabanus nigrovittatus) Wolbachia and Its Microbiome: A Preliminary Study
Source: Microbiol Spectr. 2021 Oct 13;9(2):e00517-21. doi: 10.1128/Spectrum.00517-21 (PMC8515936; doi:10.1128/Spectrum.00517-21)
Supplement: Supplemental file 1 — Supplemental material. Download SPECTRUM00517-21_Supp_1_seq8.pdf, PDF file, 1.2 MB [file spectrum00517-21_supp_1_seq8.pdf]

Supplementary Figure 1: Phylogeny of *Wolbachia* based on 4 supplementary genes.

The topology was inferred using Maximum Likelihood (ML) inference using IQTREE. Nodes are associated with bootstrap values based on 1,000 replicates, only bootstrap values superior to 70 are indicated. A) Phylogeny of *Wolbachia* based on *dnaA*. The total length of the datasets is 368bp. The Best fit model calculated using ModelFinder according to BIC index was K3Pu+I+G4. B) Phylogeny of *Wolbachia* based on *fbpA*. The total length of the datasets is 428bp. The Best fit model calculated using ModelFinder according to BIC index was K3Pu+I+G4. C) Phylogeny of *Wolbachia* based on *gatB*. The total length of the datasets is 519bp. The Best fit model calculated using ModelFinder according to BIC index was HKY+G4. D) Phylogeny of *Wolbachia* based on c*oxA*. The total length of the datasets is 211bp. The Best fit model calculated using ModelFinder according to BIC index was TPM3+G4.

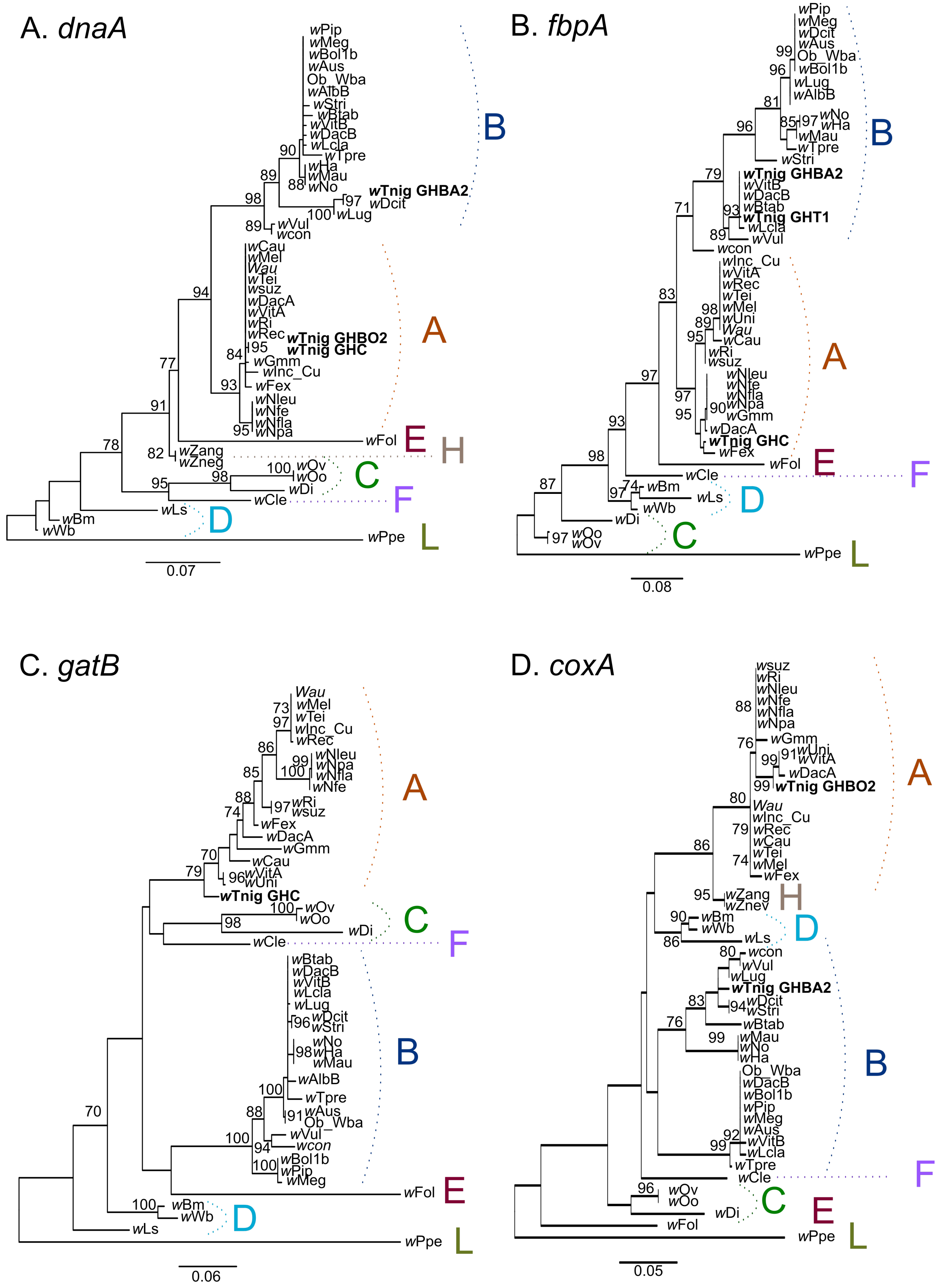

A. dnaA
B. fbpA
C. gatB
D. coxA
wTnig GHBA2
wTnig GHBO2
wTnig GHC
wTnig GHT1
A
B
C
D
E
F
H
L
0.07
0.08
0.06
0.05

Supplementary Table S1: List of accession numbers associated with sequences produced for the present study. A total of 25 PCR products were Sanger sequenced on an ABI 3730 automated DNA sequencer. Red designates *Wolbachia* supergroup A; blue designates *Wolbachia* supergroup B. negative refers to no amplification: LS refers to data with too low signal to obtain good sequencing.

	**host**	***Wolbachia***				
**specimens**	***CO1***	***ftsZ***	***dnaA***	***fbpA***	***gatB***	***coxA***
***Tabanus nigrovittatus* ind C**	**MN919540**	**MN937248**	**MN937250**	**MN937254**	**MN937257**	LS
***Tabanus nigrovittatus* ind T1**	**MN919539**	**MN937246**	negative	**MN937252**	negative	negative
***Tabanus nigrovittatus* ind T2**	**MN919545**	**MN937245**	negative	negative	LS	negative
***Tabanus nigrovittatus* ind T3**	**MN919538**	**MN937243**	negative	negative	negative	negative
***Tabanus nigrovittatus* ind BO1**	**MN919546**	negative	negative	negative	negative	negative
***Tabanus nigrovittatus* ind BO2**	**MN919544**	**MN937247**	**MN937251**	LS	LS	**MN937255**
***Tabanus nigrovittatus* ind BO3**	**MN919543**	negative	negative	negative	negative	negative
***Tabanus nigrovittatus* ind BA1**	**MN919542**	negative	negative	negative	negative	negative
***Tabanus nigrovittatus* ind BA2**	**MN919541**	**MN937244**	**MN937249**	**MN937253**	LS	**MN937256**
***Tabanus nigrovittatus* ind BA3**	**MN919547**	negative	negative	negative	negative	- negative

Supplementary Table S2: Conditions for PCR amplification used for insect and *Wolbachia* characterization.

The targeted gene, primer designations and primer sequences are listed. PCR thermal profiles listing °C temperatures (T), times in seconds (S) along with number of cycles (n) are shown. Master mix conditions included final $MgCl_2$ concentrations, buffer concentration, mM each dNTP nucleotide, µM concentration of each primer and units (U) of OneTaq® HS™ enzyme. The amplicon size is also provided.

	**Primers**		**Thermal profile**							**Master mix condition**					
**targeted gene**	**name**	**sequence**	**step1**		**step2**		**step3**		**n**	**$MgCl_2$**	**buffer**	**primers**	**dNTPs**	**OneTaq HS**	**amplicon size (bp)**
			T	S	T	S	T	S		(mM)	(X)	(µM)	(µM)	(U)	
coxI	LCO1490	GGTCAACAAATCATAAAGATATTGG	94	30	42	45	68	90	38	2.5	1	0.5	0.3	0.625	~650
	HC02198	TAAACTTCAGGGTGACCAAAAAATCA													
dnaA	wb_dnaAF1/	TCGTGGTAGGAAAGCCAAATGA	94	30	60	45	68	90	38	3	1	0.5	0.2	0.625	~910
	wb_dnaAR1	ACTGCRTGTATAACAGTAGC													
	dnaA2F/	ACAATTGGTTATATCAGCTG	94	30	52	45	68	70	40	1.5	1	0.5	0.2	0.625	~450
	dnaA2R*	TACATAGCTATTTGYCTTGG													
ftsZ	wb_ftsZF3/	GCAAATACYGATGCTCARGC	94	30	62	60	68	90	38	2	1	0.5	0.2	0.625	~860
	wb_ftsZR3	ATCAATRCCAGTTGCAAGAA													
	wb_ftsZF4/	CTAAGGGDCTTGGTGCTGGT	94	30	52	45	68	70	38	2	1	0.5	0.2	0.625	~700
	wb_ftsZR4*	ACYTCTTCRCGCACTCTATT													
fbpA	wb_fbAF1/	CATTATCATTTTCARCTTGC	94	30	59	60	68	90	38	2	1	0.5	0.2	0.625	~550
	wb_fbAR1	TCACCACCAGARAAAACTA													
	fbpAF1/	GCTGCTCCRCTTGGYWTGAT	94	30	59	45	68	70	38	1.5	1	0.5	0.2	0.625	~500
	fbpAR1*	CCRCCAGARAAAAYYACTATTC													
coxA	coxAF1/	TTGGRGCRATYAACTTTATAG	94	30	58	60	68	90	38	1	1	0.5	0.2	0.625	~850
	wb_coxAR1	TAATCAGGTATRCGYCTTGGCAT													
	coxAF1/	TTGGRGCRATYAACTTTATAG	94	30	53	45	68	70	38	1.5	1	0.5	0.2	0.625	~480
	coxAR1*	CTAAAGACTTTKACRCCAGT													
gatB	wbfilgatB_F1/	TAGATYTAAATCGTGCAGG	94	30	55	60	68	90	38	2	1	0.5	0.2	0.625	~760
	wbfilgatB_R1	TCAAACATAATATCRAAWAC													
	wbgatBF2	ATGAAAAARTTRAGGCAGATT	94	30	52.0	45	68	70	38	3	1	0.5	0.2	0.625	~600
	wbgatBR2*	GCRTTTGCTTTRAYTGGRGAG													

Supplementary Table S3: List of Wolbachia genomes (or sequences) used in the study with their strain name, NCBI accession number.

Wolbachia	strain	accession number
Wolbachia from *Operophtera brumata*	Ob_Wba	JYPC00000000
Wolbachia from *Aedes albopictus*	wAlbB	CP031221
Wolbachia from *Drosophila simulans wAu*	wAu	LK055284
Wolbachia from *Plutella australiana wAus*	wAus	MRWX00000000
Wolbachia from *Brugia malayi*	wBm	NC_006833
Wolbachia from *Hypolimnas bolina*	wBol1b	CAOH00000000
Wolbachia from *Bemisia tabaci*	wBtab	CP016430
Wolbachia from *Carposina sasakii*	wCauA	CP041215
Wolbachia from *Cimex lectularius*	wCle	AP013028
Wolbachia from *Cylisticus convexus*	wcon	QPIP00000000
Wolbachia from *Dactylopius coccus*	wDacA	LSYX00000000
Wolbachia from *Dactylopius coccus*	wDacB	LSYY00000000
Wolbachia from *Diaphorina citri*	wDcit	NZ_KB223536
Wolbachia from *Dirofilaria (Dirofilaria) immitis*	wDimm	CP046578
Wolbachia from *Formica exsecta*	wFex	RCIU00000000
Wolbachia from *Folsomia candida*	wFol Berlin	CP015510
Wolbachia from *Glossina morsitans morsitans*	wGmm	AWUH00000000
Wolbachia from *Drosophila simulans*	wHa	NC_021089
Wolbachia from *Drosophila incompta*	wIncCu	CP011148
Wolbachia from *Leptopilina clavipes*	wLcla	QJHA00000000
Wolbachia from *Litomosoides sigmondontis*	wLsig	CP046577
Wolbachia from *Nilaparvata lugens*	wLug	MUIY01000000
Wolbachia from *Drosophila mauritiana*	wMau	CP034334
Wolbachia from *Chrysomya megacephala*	wMeg	CP021120
Wolbachia from *Drosophila melanogaster*	wMel	NC_002978
Wolbachia from *Nomada ferruginata*	wNfe	LYUY00000000
Wolbachia from *Nomada flava*	wNfla	LYUW00000000
Wolbachia from *Nomada leucophthalma*	wNleu	LYUV00000000
Wolbachia from *Drosophila simulans*	wNo	NC_021084
Wolbachia from *Nomada panzeri*	wNpa	LYUX00000000
Wolbachia from *Nasonia oneida*	wOneA1	QESS00000000
Wolbachia from *Onchocerca ochengi*	wOo	NC_018267
Wolbachia from *Onchocerca volvulus*	wOv Cameroon	HG810405
Wolbachia from *Culex quinquefasciatus*	wPip	NC_010981
Wolbachia from *Pratylenchus penetrans*	wPpe	MJMG01000000
Wolbachia from *Drosophila recens*	wRec	JQAM00000000
Wolbachia from *Drosophila simulans wRi*	wRi	NC_012416
Wolbachia from *Laodelphax striatella*	wstri	MUIX00000000
Wolbachia from *Drosophila suzukii*	wsuz	CAOU00000000
Wolbachia from *Drosophila teissieri*	wTei	VCEG01000000
Wolbachia from *Trichogramma pretiosum*	wTpre	CM003641
Wolbachia from *Muscidifurax uniraptor*	wUni	MUJL00000000
Wolbachia from *Nasonia vitripennis*	wVitA	MUJM00000000
Wolbachia from *Nasonia vitripennis*	wVitB	AERW00000000
Wolbachia from *Armadillidium vulgare*	wVulC	ALWU00000000
Wolbachia from *Wuchereria bancrofti*	wWb	NJBR00000000
Wolbachia from *Zootermopsis nevadensis*	wZnev	AY764280; FJ390249; AY764284; AY764276
Wolbachia from *Zootermopsis angusticollis*	wZang	AY764279; FJ390248; AY764283; AY764275

Supplementary Table S4: Pairwise percentage similarity of the COI marker gene between *Tabanus nigrovittatus* specimens.

Similarities were calculated as percentage of identical bases divided by the total number of bases using Geneious software.

	T3	T1	C	BA1	BA2	BO3	BO2	T2	BO1	BA3	KT381971
***Tabanus nigrovittatus* ind T3**		99.9	99.9	99.9	99.9	99.9	99.8	99.7	99.3	98.4	96.3
***Tabanus nigrovittatus* ind T1**	99.9		99.8	99.8	99.8	99.8	99.8	99.6	99.2	98.4	96.2
***Tabanus nigrovittatus* ind C**	99.9	99.8		99.9	99.9	99.8	99.8	99.6	99.3	98.4	96.2
***Tabanus nigrovittatus* ind BA1**	99.9	99.8	99.9		99.9	99.8	99.8	99.6	99.3	98.4	96.2
***Tabanus nigrovittatus* ind BA2**	99.9	99.8	99.9	99.9		99.8	99.8	99.6	99.3	98.4	96.2
***Tabanus* nigrovittatus ind BO3**	99.9	99.8	99.8	99.8	99.8		99.8	99.6	99.3	98.5	96.2
***Tabanus nigrovittatus* ind BO2**	99.8	99.8	99.8	99.8	99.8	99.8		99.5	99.3	98.5	96.1
***Tabanus nigrovittatus* ind T2**	99.7	99.6	99.6	99.6	99.6	99.6	99.5		99.3	98	96
***Tabanus nigrovittatus* ind BO1**	99.3	99.2	99.3	99.3	99.3	99.3	99.3	99.3		98.2	95.4
***Tabanus nigrovittatus* ind BA3**	98.4	98.4	98.4	98.4	98.4	98.5	98.5	98	98.2		94.7
***Tabanus nigrovittatus* KT381971**	96.3	96.2	96.2	96.2	96.2	96.2	96.1	96	95.4	94.7	

Supplementary Table S5: Percentage of each class determined for four samples, BA2, BA3, BO2, BO3.

W+ refers to *Wolbachia* presence; W- refers to *Wolbachia* absence, based upon PCR analysis.

		**W+**	**W-**	**W+**	**W-**
**Class**	**sequencing technology**	**BA2**	**BA3**	**BO2**	**BO3**
Gammaproteobacteria	Illumina	40%	36%	73%	71%
	PacBio	34%	79%	63%	64%
Alphaproteobacteria	Illumina	21%	4%	25%	1%
	PacBio	44%	11%	37%	>1%
Mollicutes	Illumina	33%	57%	2%	>1%
	PacBio	16%	9%	>1%	>1%
Bacilli	Illumina	5%	2%	>1%	28%
	PacBio	6%	1%	>1%	35%

Color code:

< 0.01%	1 to 15%	16 to 30%	31 to 60%	up 60%
